# Perceptions and attitudes toward COVID-19 vaccination among health professional students in Australia: a qualitative study

**DOI:** 10.1057/s41271-024-00483-4

**Published:** 2024-04-09

**Authors:** Yingyan Chen, Marion Tower, Peta-Anne Zimmerman, Janice Layh, Vanessa Sparke, Roslyn Prichard, Matt Mason, Frances Fengzhi Lin

**Affiliations:** 1 School of Health, University of the Sunshine Coast, Sunshine Coast, Australia; 2 School of Health and Human Sciences, Southern Cross University, Gold Coast, Australia; 3 School of Nursing and Midwifery, Griffith University, Nathan, Australia; 4 School of Nursing, Midwifery & Social Work, The University of Queensland, Brisbane, Australia; 5 School of Nursing and Midwifery, Griffith University, Southport, Australia; 6 Nursing and Midwifery, College of Healthcare Sciences, James Cook University, Cairns, Australia; 7 Sunshine Coast Health Institute, Sunshine Coast, Australia; 8 College of Nursing and Health Sciences, Flinders University, Adelaide, Australia; 9 Caring Futures Institute, Flinders University, Adelaide, South Australia Australia; 10 University of the Sunshine Coast, 90 Sippy Downs Dr, Sippy Downs, Queensland 4556 Australia

**Keywords:** Students, Health professionals, COVID-19 vaccination, Perceptions, Attitudes, Australia

## Abstract

Using two qualitative data sources: free-text responses to an open-ended question of an online survey and subsequent interviews and focus groups, we explored perceptions and attitudes toward COVID-19 vaccination among health professional students enrolled in Australian universities during the pandemic with data collected from October 2021 to April 2022. Students provided free-text responses to the open-ended question (*n* = 313) in the online survey and participated in interviews or focus groups (*n* = 17). Data analysis revealed three themes, including *perceptions of COVID-19 seriousness and the risk of contracting the virus*, *information dissemination*, and *attitudes toward the vaccine mandate.* The study identified evolving perceptions of COVID-19 seriousness among Australian health professional students and their sentiments toward the vaccine mandate. There is a need to ensure the quality of information dissemination related to the vaccine mandate. This may not only support students’ uptake of mandatory vaccination but also provide a means for them to address vaccination with healthcare consumers and patients.

**Supplementary Information:**

The online version contains supplementary material available at 10.1057/s41271-024-00483-4.

## Key messages

- Perceptions of COVID-19 seriousness evolved over time.
- Participants reported that government information was overwhelming and confusing.
- Many participants maintained anti-mandate attitudes, and some criticised the mandate as “a bit double standard” in vaccine requirements between students and staff in clinical settings.
- There is a need to ensure the quality of information dissemination related to the vaccine mandate.

## Introduction

COVID-19 had claimed over 6.6 million lives and had infected more than 649 million people worldwide as of 20 December 2022 [1]. Vaccination is one of the most effective strategies to reduce the severity of illness and the associated burden of COVID-19 [2, 3]. The success of any vaccination program depends not only on vaccine availability and accessibility but also on individual acceptance to be vaccinated. Vaccine hesitancy has become increasingly common, defined as people’s resistance or refusal to be vaccinated despite the availability of the vaccine [4].

On 21 January 2020, the Parliament of Australia declared COVID-19 as a listed human disease under the Biosecurity Act 2015 [5], and on 11 March 2020, the World Health Organization declared COVID-19 a pandemic disease [6]. Prior to September 2020, the Australian Government responded to COVID-19 through travel restrictions, international border controls, and quarantine arrangements through its unique location that covers an entire continent, and we call this period the early pandemic. At the time of the data collection in October 2021 to April 2022, Australia had four vaccines approved to use, including Pfizer (since 25 January 2021), AstraZeneca (since 15 February 2021), Moderna (since 9 August 2021), and Novavax (since 19 January 2022) [7]. The Australian Government launched the COVID-19 vaccine rollout on 22 February 2021 with the national rollout strategy of providing the Pfizer vaccine to individuals in Phase 1a (priority population such as frontline healthcare workers) [7]. The Australian-made AstraZeneca vaccine was made available in March 2021, but on 8 April 2021, Pfizer was deemed as the preferred vaccine for eligible people under 50 years due to reporting a rare but potentially serious side effect of AstraZeneca [7]. But on 17 June 2021, the Australian Technical Advisory Group on Immunisation recommended Pfizer as the preferred vaccine for people 60 year old and younger. The change in advice on AstraZeneca resulted in a limited supply of Pfizer for this segment of population until September 2021, while at the same time, the Australian Government encouraged the use of existing stocks of AstraZeneca [7]. On 28 June 2021, the Australian Government introduced mandatory COVID‐19 vaccinations for workers in residential aged-care facilities, with all staff required to receive their first dose by 17 September 2021 [8]. From 11 November 2021, all health professional students were required to be fully vaccinated against COVID-19 (two doses) to attend clinical placement in all states and territories of Australia [9]. There were 5940 deaths due to COVID-19 by 30 April 2022 registered through the Australian Bureau of Statistics [10].

Research has identified vaccine hesitancy towards COVID-19 vaccination among health professional students, who are no exception to this phenomenon [11–13]. Further understanding of factors shaping their views is needed to address barriers to enhancing population-wide vaccination. Our previous nationwide online survey [9] found that approximately one in five health professional students doubted COVID-19 seriousness, perceived themselves to be at low risk for acquiring COVID-19, and disbelieved the safety of the COVID-19 vaccines. The aim of this study was to gain a deeper understanding of Australian health professional students’ perceptions and attitudes toward COVID-19 vaccination. This paper reports the qualitative component of a larger two-phase study. Phase I was a nationwide cross-sectional survey, which included quantitative items and one open-ended question at the end of the survey. We published the quantitative results elsewhere [9].

## Methods

### Study design

This is an exploratory qualitative study. Informed by results from Phase 1 survey, we conducted individual interviews and focus groups to gain an in-depth understanding of perceptions and attitudes toward COVID-19 vaccination among health professional students. A guide for interviews and focus groups was developed guided by the Theoretical Domains Framework (TDF) [14] (see Supplementary Table S1). This framework, the TDF, contains 14 domains and has been used commonly in implementation research to help identify perceived barriers and facilitators that subsequently guide behaviour change [14, 15]. We chose a theory-based framework to assist in understanding better the determinants of current and desired COVID-19 vaccination behaviours and how these determinants might be targeted from theory-based investigation to intervention [15]. An example of questions about the knowledge domain in the guide for interviews and focus groups was: “How serious do you think COVID-19 is?”.

In this paper, we also report the findings from the data from the open-ended question in Phase I survey. We followed the Consolidated criteria for reporting qualitative research guidelines when writing this paper (COREQ) [16].

### Participants and recruitment

In Phase I, we recruited 1114 health professional students from 17 Australian universities via email through our professional networks. Participants were above 18 years of age, currently resided in Australia and enrolled in an Australian university program that leads to registration with the Australian Health Practitioner Regulation Agency. More details about participants and recruitment appear in our previous publication [9].

At the end of Phase I survey, we asked interested participants to enter their contact details on a separate survey page so we could contact them in Phase II. We did not link this new page to their previous survey responses. Of 1114 health professional students who completed the survey in Phase I, 32 (2.9%) participants expressed interest in participating in Phase II by entering their contact details at the end of the survey in Phase I. The first author (YC) approached the 32 participants by email to invite them to participate in Phase II, and 17 agreed and were available to participate. The email contained a brief description of the research and a survey link where participants were able to indicate their availability. We required participants to read the participant information sheet and click on ‘Yes, consent to the study’. The participants then indicated their availabilities for further contact and data collection.

### Data collection

At the end of Phase I survey, conducted from October 2021 to January 2022, participants were asked one open-ended question: “Is there anything else you would like to say about your experiences as a health professional student related to the COVID-19 pandemic and/or COVID-19 vaccination?”. They did so with free text without a word limit.

In Phase II, we conducted one-on-one individual interviews with participants who were not available to participate in focus groups and focus groups via ZOOM (version 5.12.9) from 23 March to 6 April 2022. We included a maximum of six participants in each group for effective online facilitation. Each interview lasted up to 30 min and each focus group lasted 60 min.

Authors with previous experience in qualitative research conducted the interviews (YC) and focus groups (PZ, VS, JL, RP, and YC). These authors did not have direct working relationships with participants to prevent any power imbalance. One member facilitated each focus group while another observed. The research team asked participants to complete the demographic datasheet at the start of the interviews or focus groups. We performed member-checking [17] at the end of the interviews and focus groups to ensure what the researcher summarised was what the participant(s) had said, therefore validating the accuracy and credibility of data interpretation. We corrected any discrepancies at the end of each interview or focus group. We continued to conduct both means of collecting data until we reached data saturation [18]. Interviews and focus groups were recorded using the ZOOM program recording function and transcribed verbatim.

### Data analysis

We checked transcripts for accuracy before importing into NVivo® QSR 11, which is a software to help manage data analysis. We analysed the data of the free-text responses of the survey and transcripts of interviews and focus groups using an inductive thematic analysis approach through data familiarisation, coding, and theme identification and refinement [19]. Two of us (YC and JL) conducted data analysis on the free-text responses, and two others (MT and PZ) analysed the interview and focus group data. Two teams conducted the analysis independently using the phases of analysis to establish trustworthiness [20]. First, one author of each team read the data line by line in full several times until they were thoroughly familiar with and understood the data. Then, one author of each team categorised data into codes and sub-codes before grouping these thematically and inductively. The other authors of the teams checked all codes, sub-codes, and themes. A fifth author (FL) helped resolve disagreements during the process. The full research team discussed the generated themes and sub-themes during regular meetings and made revisions.

### Study rigour

Although inter-coder reliability is sometimes perceived as beneficial for qualitative studies [21], we applied the concepts of trustworthiness in qualitative research described below [22]. The TDF guided all steps in this qualitative study, including how we selected the study design and the sampling strategy, developed the interview and focus group guide, and collected and analysed data [14]. To enhance the credibility of the study findings, we obtained participant confirmation at the end of each interview or focus group and used participant statements as direct quotes in the results section. We acknowledged our thoughts, prior experiences, and beliefs that could influence the research process to promote reflexivity and maintain confirmability [22]. To increase the transferability of our findings, we enrolled participants who were students at 17 universities and from varied health professional disciplines. For dependability, we maintained an audit trail of all decisions we made about the research process and analysis.

### Ethics

The University of the Sunshine Coast Human Research Ethics Committee approved the study (Ethics Number A211644). We ensured that all participants learned that participation was entirely voluntary, and we provided each with a summary of information about the study before asking each to sign the written consent.

## Findings

A total of 313 participants made free-text responses to the open-ended question in Phase I survey, 10 participated in interviews, and seven in focus groups (3–4 participants in each group). We did not collect identifiable demographic data for participants as we conducted an anonymous survey, therefore, we are not able to present the demographic data for the 313 participants. From the participants in interviews and focus groups, most were female (*n* = 13; 76.5%), with a median age of 33.5 years (interquartile range 21.75–38.75). Half planned to complete their program of study within one year (Table 1). Data analysis revealed four themes. We present the themes and sub-themes with supportive quotes below, summarise them in Table 2, and illustrative quotes in Table 3.

**Table 1:** Participant characteristics (*N* = 17)

	No. (%)
Age (*n* = 14)* median (IQR)	32.5 (21.75–38.75)
Gender	
Female	13 (76.5)
Male	4 (23.5)
Cultural background	
Australia	12 (70.6)
British	2 (11.7)
Australian and Chinese	1 (5.9)
South America	1 (5.9)
Brazilian	1 (5.9)
Current program of study	
Nursing	8 (47.1)
Midwifery	4 (23.4)
Occupational therapy	2 (11.8)
Psychology	1 (5.9)
Paramedicine	1 (5.9)
Medicine	1 (5.9)
How far toward completion	
Less than 1 year	5 (29.4)
1–2 years	5 (29.4)
More than 2 years	7 (41.2)
Plan to complete study	
Less than 1 year	9 (52.9)
1–2 years	5 (29.4)
More than 2 years	3 (17.7)
Carer responsibility outside of study	
Yes	10 (58.8)
No	7 (41.2)

*IQR* Interquartile range

An asterisk * indicates the number of participants with data available

**Table 2:** Themes and sub-themes

Themes	Sub-themes
Perceptions of COVID-19 seriousness and the risk of contracting the virus	Perception of COVID-19 seriousness
	Perception of the risk of contracting the virus
Information dissemination	Trust in research evidence on vaccine safety
	Clarity of communication
	Sources of information
Attitudes toward vaccine mandate	Anti-mandate
	Pro-mandate
	“A bit double standard”

**Table 3:** Illustrative quotes

Quote	Content	Description
Q1	“The amount [sic] of deaths from the flu in a given year in Australia is like a couple of 100 from memory. And, like, from COVID, it was 1000. And the flu, generally speaking, doesn’t give you any long-term complications, whereas COVID does.”	PPT 3, FG 4
Q2	“I think initially, it was very serious because we didn’t really understand like, how the virus was going to go and how it was going to spread. But I think as the vaccinations have come out and we’ve had more education on what COVID is and how it is, like how we’re responding to it. And I think the look of how serious it is, is actually kind of gone down quite a lot.”	PPT 3, FG 3
Q3	“… not necessarily more or less than anyone else. I think some of the precautionary measures that we have in place mean that it’s probably about the same as it is for anyone else.”	INT, PPT 1
Q4	“Um, I definitely think that the risk is higher. Because we're exposed a lot more to potentially hospital settings, so you have a lot more sick people. And you're also maybe not taking the social distancing precautions when you're attending to people.”	PPT3, FG 2
Q5	“I think it’s because it was a global corporation working towards this vaccine for me this is clear that that’s why we got this vaccine… And for me, this is no issue [sic].”	PPT 5, FG 1
Q6	“The hospitals were calling students and saying you can come back now. And the students were going, I can’t because xxx [their university] hasn’t said that I can”	INT, PPT 1
Q7	“We haven’t been given any choice to exercise our human right to choose what we think is safe to be placed inside our own bodies. This is an abomination of the human race and their rights to choose and say yes or no. I am disgusted ... for how we are dealing with this issue and how we are getting STRIPPED of our rights.”	Anonymous, Free-text responses
Q8	“It just was a terrible choice (especially since I have underlying health conditions) it felt like I had to choose between potentially destroying my health or following my dream job. I picked the latter and felt like I gambled with my health.”	Anonymous, free-text responses
Q9	“If you’re, you know, offered some paracetamol and you say, “No, thank you, I don’t want it”. Well, that’s fine. But you’re going to have pain for the next four hours…you do have the right to choose, but it is going to have consequences.”	INT, PPT 1
Q10	“… disappointed that although it was made mandatory for students not to attend clinical placement unless we had the vaccine, this was not implemented by xxx [state] Health until months later for their own staff.”	Anonymous, free-text responses

*PPT* participant, *INT* interview, *FG* focus group

### Perceptions of COVID-19 seriousness and the risk of contracting the virus

This theme describes how participants perceived COVID-19 seriousness and their risks of contracting the virus. Most perceived COVID-19 as a serious illness, particularly when so little was known about it in the early stages of the COVID-19 pandemic (e.g., between January and September 2020). They believed COVID-19 was more serious than other viruses, such as seasonal influenza (Table 3, Q1).

Perception of COVID-19 seriousness also evolved with time. Participants described that as more became known about COVID-19, they perceived COVID-19 to have been less serious, especially with the implementation of vaccination (Table 3, Q2). When asked about their risk of contracting the virus, many participants believed that they had the same or lower risk than the general population. Participants reported that their lower risk derived from the additional measures taken in healthcare facilities to protect staff, for example, masks, other personal protective equipment, and swabbing of patients [for PCR test] (Table 3, Q3). In addition, some participants believed that younger age protected them from severe disease; however, not all participants agreed.

A small number of participants believed they were at greater risk because they would be exposed to the virus more often than the general public while on clinical placements (Table 3, Q4). Some participants also noted that because they were still learning, they could make mistakes with infection prevention and control precautions that might make them vulnerable—for example, forgetting to perform hand hygiene.

### Information dissemination

This theme relates to the perceptions of and attitudes about information dissemination that influenced COVID-19 vaccine uptake and includes three sub-themes: trust in research evidence on vaccine safety, clarity of communication, and sources of information.

Most participants trusted research evidence on vaccine safety. They expected side effects with the implementation of any new vaccine and reported those side effects to be preferable to acquiring the virus. They drew confidence in the safety and efficacy of the vaccine from the scientific processes underpinning its development. While they acknowledged that the speed at which the vaccine had been released was a source of anger for many people, most participants related this to the investment of resources in securing a COVID-19 vaccine compared to other vaccines (Table 3, Q5).

Regarding the clarity of communication, most participants stated that their universities had provided clear information to students about the vaccine requirements. However, this clear communication and subsequent trust the students garnered from their teaching institutions were frequently undermined by the changes and inconsistency in rules issued by State and Federal health departments, as stated by several participants. This resulted in confusions related to mandatory vaccine requirements, where students must have been vaccinated to be able to attend clinical placements. The confusions were perceived to be related to communication delays between healthcare organisations and the university (Table 3, Q6). As a result, some students lost their clinical placements.

Also, participants held mixed views about trusted sources of information, such as government websites. Some reported feeling confused and overwhelmed by the volume or frequently changing information available on government websites. Several participants noted approaching government websites cautiously, suggesting that the information provided might be about furthering government propaganda, which could fuel distrust of the vaccination.

### Attitudes toward vaccine mandate

This theme describes participants’ perceptions of and attitudes toward the vaccine mandate, including sub-themes of anti-mandate and pro-mandate. Anti-mandate is the most common sub-theme coming from the free-text responses (Table 3, Q7). The mandate means that health professional students were required to have been vaccinated to be able to continue clinical placements.

Some participants wanted to be clear that anti-mandate should not be automatically labelled as ‘anti-vax’. The government claimed the vaccine mandate was voluntary, but students must receive vaccines as per the vaccine mandate to continue their clinical placement. This made the students feel that they were not given the autonomy to choose. Some participants also felt the vaccine mandate led them to linger amidst potentially conflicting choices for career, freedom, and health (Table 3, Q8). Most participants commented that people still had the choice to be vaccinated or refuse the vaccine. However, people should accept the consequences of their decision. One participant likened this to declining a pain medication (Table 3, Q9).

In contrast, some participants agreed with the COVID-19 vaccine mandate because they perceived that the COVID-19 vaccine was no different from other mandatory vaccines for health professional students. Being vaccinated against COVID-19 was a professional responsibility. Participants criticised those health professional students who were anti-mandate for “*making such a big deal over nothing*” [Anonymous, free-text responses].

Participants criticised the vaccine mandate policy “*a bit double standard*” [Anonymous, free-text responses]. They stated that the mandate was imposed earlier for students than for staff. They were informed that they had to be double vaccinated to attend clinical placements with just 24 h’ notice, and they were removed from their clinical placements because of lockdowns when they were fully vaccinated. At the same time, healthcare employees were not all vaccinated and even those who were now still working (Table 3, Q10).

## Discussion

This study explored Australian health professional students’ perceptions and attitudes toward COVID-19 vaccination. Health professional hesitancy in vaccination can undermine public confidence in COVID-19 vaccination and potentially result in life-threatening illness in unvaccinated persons [23].

The perception of COVID-19 seriousness can influence vaccination behaviour [9, 24, 25]. In our study, most participants perceived COVID-19 as a serious disease in contrast with previous research where participants claimed that COVID-19 was less serious than was said and even denied the presence of a pandemic [9, 25, 26]. The perception of COVID-19 seriousness may evolve depending on personal experience with COVID-19 and the level of pandemic severity as people may rush to be vaccinated if the pandemic becomes more severe [27, 28]. The perception of COVID-19 seriousness can evolve with time as in our study: participants perceived COVID-19 being less serious later than at the beginning, especially with the implementation of vaccination and more education about the virus. With further waves of the virus occurring, we encourage future research to examine factors that alter the association between perceived severity and intention to seek or accept a subsequent dose.

The perception of the likelihood of contracting COVID-19 infection is key in the decision-making process leading to vaccination [26, 29, 30]. In our study, most participants believed they had the same or lower risk than the general population of contracting the COVID-19 virus. This is consistent with previous research that indicated health professional students often perceive they have lower risks of infection [9, 13, 31]. Many factors may have contributed to this, such as claiming to be young and healthy without underlying health conditions and limited exposure to the COVID-19 virus [24, 30, 32]. In our study, lower risk perception was ascribed to proper personal protective equipment used during clinical placements, consistent with an earlier study [33]. Although not reported in our study, risk perception can evolve depending on the degree of threat experienced during the pandemic, such as community transmission [34, 35]. As perceptions of being at lower risk often indicate less likelihood of vaccination, we suggest that universities re-design relevant curricula by incorporating COVID-19 research evidence to increase health professional students’ perceptions of risk, including the emerging evidence of ‘long COVID-19’ [24, 36] and emphasising that virus ‘doesn’t choose’ among everyone [37].

Disseminating information to promote trust in research on vaccine safety can also influence individual’s decision-making about their vaccination intentions and behaviour [30, 34, 35, 38]. Our study findings show that most participants believed in vaccine safety, drew confidence in the vaccine development process, and accepted vaccine side effects. Many studies reported concerns about vaccine safety, a key contributor to vaccine hesitancy [25, 30, 35, 39]. Some participants in other studies expressed worries about the speed of vaccine development and suspicion of being experimental using the term ‘guinea pig’ [25, 30]. These worries lead to questions about why treatments and cures were not found for conditions, such as cancer, that have been around longer than COVID-19 [40]. The government needs to convey the message clearly, emphasising that vaccine development is a global effort [3]. Other factors, such as lack of information and evidence, could also lead to the uncertainty of vaccine safety. Brown et al*.* [39] identified four routes to resolve uncertainty, by providing access to better and more information, acknowledging that the potential unknown risk of the vaccines must have lessened, recognising that the vaccines may be the only way to contain the pandemic and regain freedom, and realising that there would be no choice but to have the vaccine because of the ‘vaccine passport’ use. Healthcare workers or scientific experts need to disseminate the vaccine information effectively so that vaccine safety concerns can be minimised, hence facilitating vaccine acceptance [29, 31].

Clear information dissemination about COVID-19 and its management from the governments is important to build people’s trust and confidence, which has been reported to increase vaccination uptake [38, 41]. Studies found that the governments made efforts to manage a very difficult situation [39] and that prioritising vaccination for frontline healthcare workers and those at high risk of infection was perceived positively [38]. However, research shows that a general distrust of the governments and the information they disseminated frequently posed a substantial barrier to people’s vaccination acceptance [2, 30, 32, 37, 42]. A qualitative study exploring attitudes of vaccine-hesitant adults in the United Kingdom revealed that many participants distrusted their government due to its ever-changing rules, guidance, and information, claiming that political agendas facilitated government actions rather than health [32]. Most participants in our study felt confused with and overwhelmed by the volume or frequently changing information from the government and this led to distrust. Therefore, it is imperative for governments to disseminate information effectively, considering information content and preferred means of access and communication for different groups of people to re-build trust and promote vaccination uptake [2, 37, 41, 42], while acknowledging the pandemic uncertainty.

Our findings revealed that participants had two different views toward the vaccine mandate. The participants we interviewed all agreed with the vaccine mandate. However, analysis of the free-text responses in our study demonstrated strong anti-mandate sentiments. In accordance with previous research [30, 38, 39, 42], our participants in the online survey expressed anger by stating that the Australian vaccine mandate for health professionals and students did not allow autonomy. Anger induced by the vaccine mandate [42] and its potential associated vaccination resistance [11, 30] highlights the importance of introducing the vaccine mandate in a planned manner, having strategies to allay potential concerns, and increasing vaccination acceptance and uptake prior to its implementation [43].

Our study illustrates three lessons learned that could be useful for the wider community. First, exploring perceptions of the COVID-19 seriousness is important to assist in identifying the barriers to vaccination, and hence, strategies can be put in place to address the identified barriers. Second, the information dissemination should be clear and simple and based on scientific evidence. Finally, when introducing the vaccine mandate policy, the government should plan it carefully by involving key stakeholders, who will assist in communicating with their teams.

## Strengths and limitations

Strengths of this study are the efforts to maintain rigour by following a theoretical framework and the analysis of both free-text responses and interview/focus group data, which have provided insight into the complexity of perceptions and attitudes toward COVID-19 vaccination among Australian health professional students. We focused on health professional students in our study because they are future healthcare workers who will play vital roles in information dissemination, vaccine administration, or both [38]. The public may identify health professional students as reliable sources for medical advice and information clarification [41]. Understanding these students’ perceptions helped identify potential barriers to promoting vaccination among them and the general public. These findings can then help identify specific areas to influence vaccination uptake amongst health professional students and influence healthcare policies in relation to vaccination campaigns and educational institutions’ initiatives. During our data collection, we also ensured that all health professional students were interviewed by our research team member(s) who were not the participants’ faculty members to reduce the power imbalance.

Limitations include potential response bias, as participants in interviews and focus groups were more pro-mandate compared to those who made free-text responses and were more anti-mandate. Social desirability bias could have occurred in all interviews and focus groups, as participants may have answered one of the questions, “What do you think about the vaccine mandate?” in a manner viewed as favourable by the researcher(s). As our participants were mainly nursing students, and because nursing professionals played a crucial role involving COVID-19 vaccination, their existing knowledge about COVID-19 may have influenced their responses regarding vaccinations. Additionally, this study was conducted in Australia, a country that has its uniqueness in relation to COVID-19 infection rates, number of deaths, and pandemic management strategies; hence, the study findings may not apply to other countries.

## Conclusions

Findings from this study provided information for policy-makers in educational institutions, healthcare organisations, and government at a local and national level to promote the COVID-19 vaccination uptake among health professional students in Australia. The findings revealed that health professional students’ perceptions of COVID-19 seriousness evolved over time, highlighting that healthcare organisations and educational institutions should assess the health professional students’ perceptions before promoting the use of the subsequent vaccine doses, therefore increasing its uptake. The findings also highlight that the government needs to utilise more effective public communication strategies to ensure the messages are clear and succinct, and information communicated needs to be based on research evidence to promote further vaccination uptake. The lessons learned from the introduction of the vaccine mandate in Australia could inform decision-making on how to respond to future health crises.

### Supplementary Information

Below is the link to the electronic supplementary material.

Supplementary file1 (DOCX 31 kb)

## Data Availability

Data will be available upon a request.
