# Supplementary material for: Perceptions and attitudes toward COVID-19 vaccination among health professional students in Australia: a qualitative study
Source: J Public Health Policy. 2024 Apr 9;45(2):283–98. doi: 10.1057/s41271-024-00483-4 (PMC11178491; doi:10.1057/s41271-024-00483-4)
Supplement: Supplementary file 1 — Supplementary file1 (DOCX 31 kb) [file 41271_2024_483_MOESM1_ESM.docx]

**Journal of Public Health Policy**

Supplementary Material

# Title: Perceptions and attitudes toward COVID-19 vaccination among health professional students: a qualitative study

Yingyan Chen ^1,2*^, Marion Tower ^3,4^, Peta-Anne Zimmerman ^5^, Janice Layh ^1^, Vanessa Sparke ^6^, Roslyn Prichard ^1^, Matt Mason ^1^, Frances Fengzhi Lin ^1,7,8^

^1^ School of Health, University of the Sunshine Coast, Sunshine Coast, Australia

^2^ School of Health and Human Sciences, Southern Cross University, Gold Coast, Australia

^3^ School of Nursing and Midwifery, Griffith University, Nathan, Australia

^4^ School of Nursing, Midwifery & Social Work, The University of Queensland, Brisbane, Australia

^5^ School of Nursing and Midwifery, Griffith University, Southport, Australia

^6^ Nursing and Midwifery, College of Healthcare Sciences, James Cook University, Cairns, Australia

^7^ Sunshine Coast Health Institute, Sunshine Coast, Australia

^8^ College of Nursing and Health Sciences, Flinders University, Australia

* Corresponding author:

Dr Yingyan Chen

[ychen@usc.edu.au](mailto:ychen@usc.edu.au)

90 Sippy Downs Dr, Sippy Downs, University of the Sunshine Coast, Queensland 4556,

Australia

Running Title: Student Perceptions on COVID-19 Vaccine

**Journal of Public Health Policy**

Supplementary Material

**Perceptions and attitudes toward COVID-19 vaccination among health professional students: a qualitative study**

Supplementary file Table S1

**Interview guide**

| **No.** | **Main questions** | **Prompt questions** | **TDF domains** | **Based-on*** |
| --- | --- | --- | --- | --- |
| 1 | How serious do you think COVID-19 is? | **(1)** More than 12% didn’t think the COVID-19 was a serious global health crisis and over 27% said that COVID-19 wasn’t more serious than the seasonal flu. What are your thoughts about this? | Knowledge | Q7, Q11 |
|  |  | **(3)** 11.8% didn’t think that the pandemic would negatively affect their life in the next 12 months. What do you think about the negative effects over the next 12 months? | Beliefs about consequences /knowledge | Q13 |
|  |  | **(4)** About 8.3% said that COVID-19 did not impact their life for the last 18 months. Why do you think some people wouldn’t feel it had impacted their life? |  | Q12 |
| 2 | What do you think is the risk of health students getting COVID-19? | **(1)** Around 32% weren’t worried about becoming seriously ill with the COVID-19. Why do you think they weren’t worried? | Emotions | Q54 |
|  |  | **(2)** About 13% of participants didn’t believe health students have a higher risk of getting COVID-19. What might make them think in that way? | Beliefs about consequences /knowledge | Q50 |
|  |  | **(3)** About 14% disagreed that the risks associated with getting the COVID-19 were higher than the ones related to getting the vaccine. Why do you think people would be more concerned about getting the vaccine as opposed to catching COVID-19? | Beliefs about consequences /knowledge | Q51 |
|  |  | **(4)** About 11% of the participants didn’t think that mask-wearing was an essential element in controlling the spread of the COVID-19. What is your opinion on this? | Knowledge | Q18 |
| 3 | Do you think currently available COVID-19 vaccines are safe? If not, why? | **(1)** 46.8% were worried about the side effects of the vaccines and about one-third were worried that the vaccine could negatively affect their health. Do you share these worries? Why? What about the vaccine’s effect on fertility and breastfeeding? Can you tell me about that? | Beliefs about consequences /knowledge | Q30, Q37, Q38, Q74 |
|  |  | **(2)** 29.4% stated that they felt the COVID-19 vaccines were developed too quickly to be safe. What are your thoughts on this? |  |  |
|  |  | **(3)** 20.3% didn’t trust that pharmaceutical companies had been required to prove that their vaccines were safe. 15.5% didn’t believe Australia’s currently available COVID-19 vaccines were safe. What do you think about the process for ensuring vaccine safety and whether the vaccines are safe? | Social context /Knowledge | Q101, Q28 |
|  |  | **(4)** 17.4% didn’t think currently available vaccines were effective at lowering the rates of transmission of the COVID-19. What do you think? | Knowledge | Q43 |
| 4 | The top three sources students trust and use for information about the COVID-19 pandemic and vaccines are official health professionals, government websites, and WHO). What is your opinion on this? | **(1)** 11.2% felt it wasn’t easy to find reliable information about the vaccines’ safety. How easy do you think it is to find reliable information? Where did you usually go to get this information? Why do you believe this information is reliable? | Context and resources | Q100 |
|  |  | **(2)** Over 22% didn’t think state/territory/federal governments were working in the best interests of their community. What is your view? Is one better than the other? | Social context | Q102, Q103 |
|  |  | **(3)** 34.6% didn’t believe information from governments was clear. Do you agree? What about information from your place of study about the requirement for vaccination? Was it clear to you (11.8% disagree)? What might have made it clearer? | Social context | Q106, Q107 |
|  |  | **(4)** Roughly 20% said that TV and social media discouraged students from getting vaccinated. How has information from TV or social media influenced people to get vaccinated? | Social context | Q104, Q105 |
| 5 | 8.8% of participants have not received a COVID-19 vaccination (Q115). Out of those people, 71.6% are not planning to get the vaccines. What do you think are the factors that influenced students’ decisions on getting vaccinated? | **(1)** About 32% agreed to wait until they know more about the long-term side effects of vaccines before being vaccinated. What is your opinion on this? | Behavioural regulation | Q79 |
|  |  | **(2)** Around 16% of participants said getting recommended or preferred vaccines and finding time for vaccination has been difficult. What was your experience? Why might it have been difficult for others? | Context and resources | Q118, Q119, Q121 |
|  |  | **(3)** Approximately 13% of the students didn’t think receiving vaccines protected the health of themselves, family, or their community. What is your view on this? | Social context | Q22, Q23, Q24 |
|  |  | **(4)** Do you think it is an ethical decision to get vaccinated? What might be some of the ethical considerations? | Professional role |  |
|  |  | **(5)** Does your studying /working environment have enough resources needed to allow you to get vaccinated? | Context and resources | May not be needed |
| 6 | The vaccine mandate has been in place for a while and received different responses. What do you think the vaccine mandate? | **(1)** 34.3% agreed that COVID-19 vaccination should not be mandatory at all. What is your view on the vaccine mandate? | Emotions | Q35 |
|  |  | **(2)** Several participants commented that this mandate has no difference from other vaccine mandates in the past. What do you think? |  |  |
|  |  | **(3)** Others commented that this mandate contradicted what they have been taught in their courses that patients have the right to choose or refuse medications. What is your opinion on this? |  |  |
|  |  | **(4)** Also, many disagreed with what they perceived as discrimination between vaxed and unvaxed. What are your thoughts about this? |  |  |
|  |  | **(5)** Last question about this mandate is “how do you feel about deciding between your career or your health because of this mandate? |  |  |
| 7 | What do you think needs to be done to improve vaccine uptake among students? | **(1)** 75.2% of students actively encouraged family and friends to get the vaccines, and 82.2% believed getting vaccinated was an important professional responsibility. Do you think encouraging others to get vaccinated is an important part of your role as a health student? | Behavioural regulation, social/ professional role and identity | Q31, Q32, Q122 |
|  |  | **(2)** Some people (over 17%) were worried about passing the COVID-19 to their family or colleagues. Do you think this worry helped encourage students to get vaccinated? | Emotions | Q53, Q55 |
|  |  | **(3)** Others (28.2%) were only vaccinated because of the mandate. Do you think the mandate played a big role in students getting vaccinated? | Social context | Q41 |

Note: * indicates which survey questions informed the interview questions.

Areas in grey are prompting questions.
